# EDITORIAL: Advances in Research on Pharmacological Targets for Pain Relief

**DOI:** 10.2174/1570159X11311060001

**Published:** 2013-12

**Authors:** Sun Wook Hwang

Several years ago, a former hot topic volume of Current Neuropharmacology assembled elegant reviews and perspectives that summarized and discussed state-of-the-art advances in research on the neurobiology of pain [1]. The current topography of the field is highlighting the need for new updates of scientific achievements and their pharmacological prospects. Viewpoints and technologies evaluating pain states have gradually evolved and novel pain target molecules are emerging. Newly discovered molecules shed light on future developments of promising pharmacological approaches. Accordingly, this issue collected highly insightful and informative reviews reflecting the many aspects of progress in the pain research.

In the first review, Dr. Enrique J. Cobos and Dr. Enrique Portillo-Salido asked essential questions regarding the translational processes in the assessment of pharmacological potencies and efficacies. They sought to revise both our focus and view on the examination of therapeutic prospects during the preclinical period by intensively discussing the validity of pain assessment systems.

Dr. Fumimasa Amaya, Dr. Yuta Izumi, Dr. Mika Sasaki, and Dr. Megumi Matsuda introduced a variety of pro-nociceptive mediators generated under injury conditions and discussed the associated mechanisms that drive pain hypersensitivity at the peripheral level and the validity of the molecular components as pharmacological targets.

In the following review, Dr. Nicolas Cenac emphasized the crucial roles of protease-activated receptors and their signal transductions in visceral pain from the gut, pancreas and bladder as well as in functional bowel disorders.

Dr. Seungkyu Lee described pain transmission and plasticity via the action of various neuronal voltage-gated calcium channels. Furthermore, for their inhibitors ranging from traditional to atypical ones, detailed molecular mechanisms and beneficial outcomes for chronic pain were summarized.

The role of potassium channels is as important as that of excitatory ion channels in nociceptive firing. Dr. Nikita Gamper and Dr. Xiaona Du highlighted potassium channels as validated pain targets by extensive examination of evidence from molecular biology, physiology, and pharmacology.

Very recently, the calcium-activated anion channel Anoctamin 1 made its debut as an important transducer for thermal pain and inflammatory mediator-induced pain. Dr. Hawon Cho and Dr. Uhtaek Oh, the researchers who first discovered this ion channel molecule, offered an in-depth analysis of its role in peripheral pain sensation.

The importance of sensory TRP channels in the pain killing strategies has been established over the course of a decade. Dr. Hongzhen Hu, Dr. Jialie Luo, Dr. Edgar T. Walters, and Dr. Susan M. Carlton provided a review concerned with major issues regarding the TRP-mediated pain and their synthetic ligands.

Resolvins and related omega-3-derived lipid substances are emerging as pro-resolving and anti-inflammatory mediators. Lastly, Dr. Sun Wook Hwang, Dr. Sungjae Yoo, and Dr. Ji Yeon Lim discussed their pain-killing aspects and druggability by reviewing recent results from analgesic assessments.

The series of excellent reviews presented in this special issue will lead to a comprehensive understanding of the current trends in pain physiology and pharmacology research. Furthermore, hopefully this volume will help readers to generate future creative pain hypotheses, and to propose practical insights aimed at developing novel painkilling strategies.

## References

[1] Gereau RW, Nicoletti F (2006). Editorial. Curr. Neuropharmacol.

